# A Pediatric Gastric Dieulafoy Lesion: A Case Report and Literature Review

**DOI:** 10.7759/cureus.55376

**Published:** 2024-03-02

**Authors:** Mário Ribeiro, Rita A Silva, Diana R Oliveira, Dália Fernandes, Filipa Neiva

**Affiliations:** 1 Pediatrics, Hospital de Braga, Braga, PRT; 2 Gastroenterology and Hepatology, Hospital de Braga, Braga, PRT; 3 Pediatrics, Unidade de Gastrenterologia Pediátrica, Serviço de Pediatria, Hospital de Braga, Braga, PRT

**Keywords:** vascular malformation, hemoclipping, gastrointestinal bleeding, gastric arterial ectasia, endoscopic treatment, dieulafoy lesion

## Abstract

This report presents a case of a 16-year-old male with severe upper gastrointestinal bleeding caused by a Dieulafoy lesion (DL). A DL is a rare but life-threatening condition characterized by sudden and massive bleeding from a small arterial vessel in the gastrointestinal (GI) tract. Diagnosis is often made through esophagogastroduodenoscopy (EGD), which reveals an enlarged submucosal blood vessel. The patient was successfully treated with adrenaline injection and hemoclipping during EGD. This case highlights the importance of considering a DL as a potential cause of severe upper GI bleeding in pediatric patients and emphasizes the significance of early recognition and intervention to achieve favorable outcomes. Additional investigation is required to enhance our comprehension of the occurrence, etiology, and most effective approaches to managing DLs in pediatric patients.

## Introduction

A Dieulafoy lesion (DL), a vascular malformation of the gastrointestinal (GI) tract, is a rare but potentially life-threatening condition that involves sudden and massive bleeding from submucosal blood vessels in the absence of any abnormality, such as ulcers or erosions in the GI tract. At a macroscopic level, this malformation is characterized by a small-sized lesion that presents as a mucosal defect, accompanied by an artery protruding from its base [1]. 

The lesion can occur anywhere along the GI tract, but it most commonly affects the stomach, particularly the lesser curvature. In adults, DLs are observed with a higher frequency in men, with a ratio of 2:1, whereas in the pediatric population, they occur equally among both genders. These lesions are reported to manifest across all age groups; however, they are most prevalent in the elderly, typically in their sixth decade of life. Patients with DLs tend to exhibit multiple comorbidities, including hypertension, chronic kidney disease, and diabetes mellitus [2,3]. The incidence of DLs in children remains undefined and they can occur at any age, which supports the proposed congenital origin of the disease [3].

The etiology and pathophysiology of DLs are poorly understood. However, the consensus is that ischemic injury, probably related to comorbidities or drugs such as non-steroidal anti-inflammatory drugs (NSAIDs) and antithrombotic drugs, leads to the disruption of the overlying epithelium and abnormal vascular development, resulting in the formation of an abnormally large arterial vessel in the submucosal layer of the GI tract. This vessel is fragile and can easily rupture, leading to massive bleeding. Patients with DLs often present with sudden and severe GI bleeding, which can be life-threatening if left untreated. The bleeding can be recurrent and can lead to chronic anemia, requiring repeated blood transfusions [4].

This report presents a young patient with severe upper GI bleeding due to a DL in the stomach, which was diagnosed by esophagogastroduodenoscopy (EGD) and successfully treated with hemoclipping.

## Case presentation

A 16-year-old male, with unremarkable personal medical and surgical history, was admitted to the emergency department with a chief complaint of severe massive hematemesis that occurred one hour before admission. He also complained of fatigue, dizziness over the previous four days, and one episode of syncope lasting between 15 and 30 seconds. Both parents did not mention any trauma, medication (such as NSAIDs), or eliciting factors that could have led to the symptoms. On physical examination, he was pale, apyretic, and hemodynamically stable. His abdomen was soft, non-tender, and non-distended. No neurological abnormalities were noted. Initial laboratory results showed normocytic, normochromic anemia (hemoglobin of 6.7 g/dL), with a normal white blood cell count and a platelet count of 181.000/mm^3^. Prothrombin and partial thromboplastin times were also normal.

**Figure 1:**
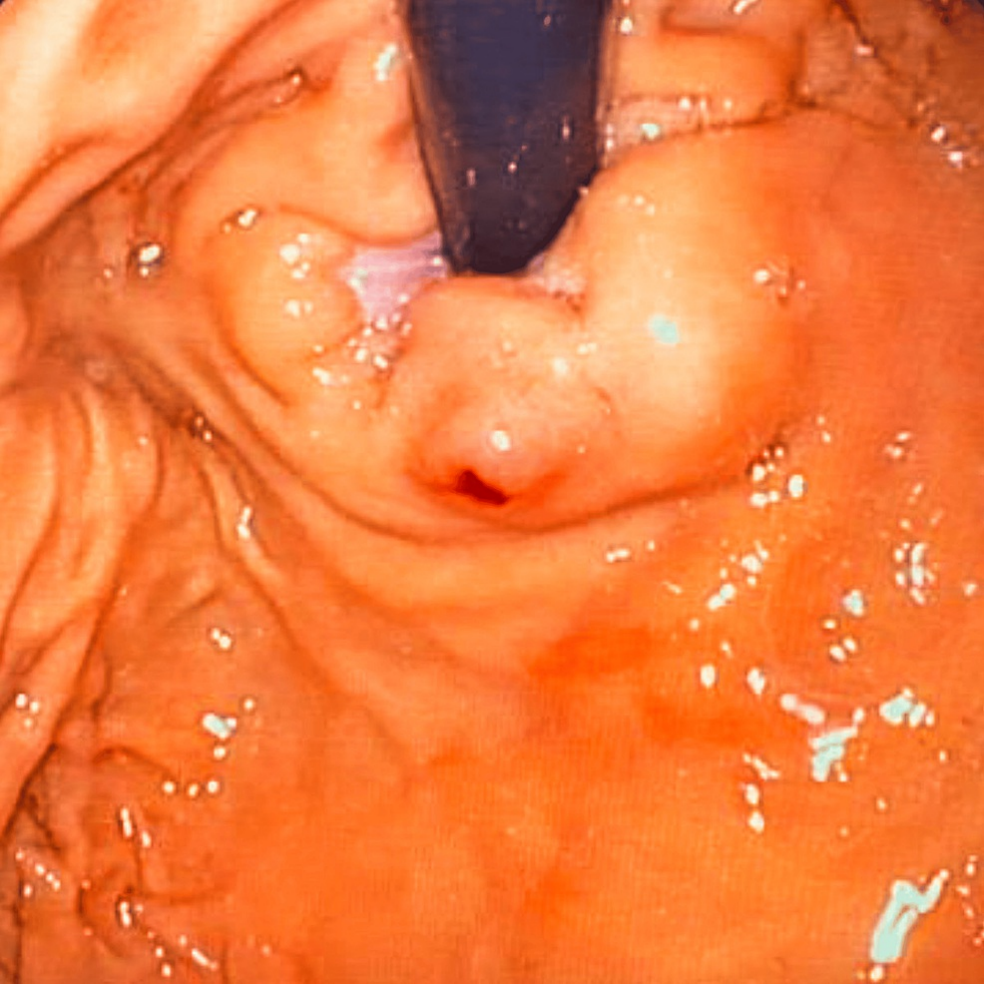
Dieulafoy lesion in the gastric fundus.

**Figure 2:**
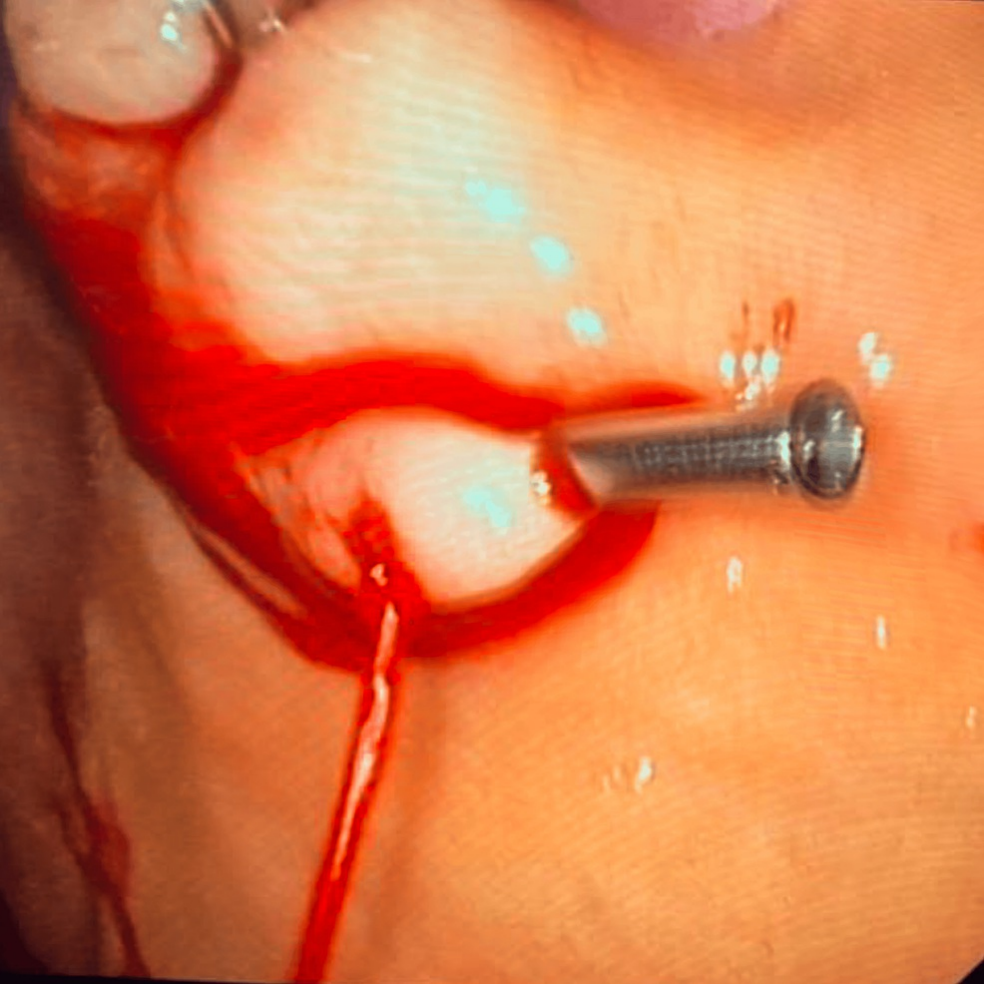
Jet hemorrhage after initial hemoclipping.

**Figure 3:**
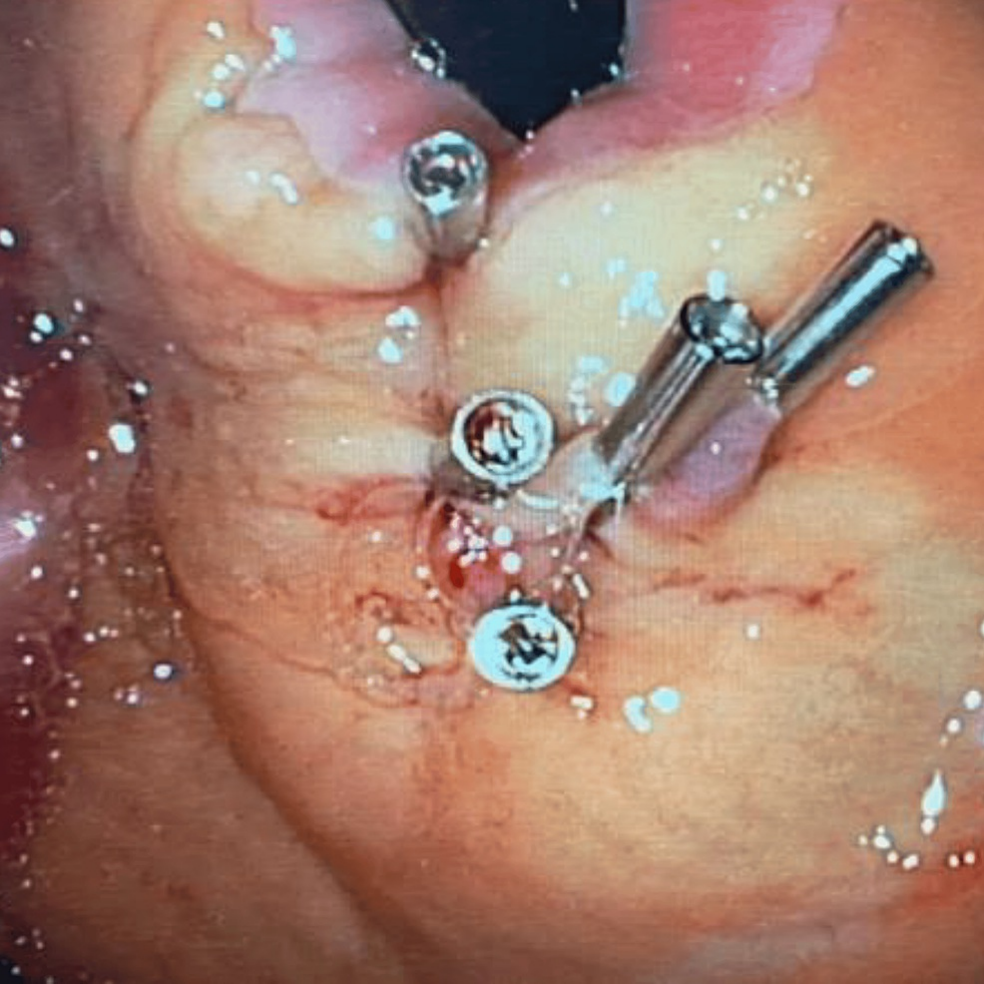
Hemoclipping was performed on the exposed vessel.

The patient’s anemia associated with hematemesis raised the main diagnostic hypothesis of upper GI bleeding. After initial observation, he presented melena and had a syncopal episode. The patient was kept nil by mouth, a unit of packed red blood cells was transfused, and he was treated with proton pump inhibitor infusion (pantoprazole 8 mg/hour). After an initial improvement of clinical status and laboratory parameters, the patient presented a sudden deterioration due to another syncopal episode, with new laboratory exams showing worsening anemia with a drop in hemoglobin to 5.9 g/dL. Emergency EGD revealed brisk bleeding from an enlarged submucosal blood vessel in the gastric fundus (Figure 1), which was consistent with a DL. This suspicion was confirmed by the jet hemorrhage triggered after the placement of the first hemoclip (Figure 2). The DL was treated with 3 ml injection of adrenaline (1:10000) and hemostasis was achieved through the application of clips (Figure 3) onto the lesion.

The patient was transferred to the pediatric intermediate care unit (ICU), oral iron treatment was initiated (200 mg daily), and he was allowed to take orally within the next 24 hours. He did not experience any other episodes of hematemesis, dizziness, syncope, or fatiguability after successful therapy. Five days after ICU admission, new laboratory results revealed a rise in hemoglobin levels to 10.3 g/dL. The patient was discharged on oral iron supplementation, 10 days after admission. At the follow-up appointment four weeks later, a new blood test showed a hemoglobin level of 13.5 g/dL.

## Discussion

The presented case report highlights a rare but potentially life-threatening condition in a pediatric patient. The clinical manifestation of DLs displays variability based on the lesion's location. Gastric or duodenal lesions typically manifest as significant upper GI bleeding. Those in the small intestine may present with upper GI bleeding or hematochezia, while colonic lesions tend to present also with hematochezia or occult bleeding. Other GI locations include the esophagus and rectum. In rare instances, extragastrointestinal lesions have been documented in the bronchial tree [5].

Diagnosis is often made via direct endoscopic visualization. The endoscopic diagnostic criteria for DL include active arterial spurting or micropulsatile streaming from a minute mucosal defect (< 3 mm); direct visualization of the protruding vessel with or without active bleeding within the mucosal defect with normal mucosa along the periphery; evidence of a densely adherent clot with a narrow attachment point to a mucosal defect or normal-appearing mucosa [6].

However, in some cases, the diagnosis can be challenging, as the lesion is often small or intermittent and can be difficult to detect during endoscopic examination (especially when endoscopy is not performed at the time of hemorrhage). Imaging studies, such as computed tomography (CT) scans or angiograms, may be helpful in identifying the lesion and determining the source of bleeding.

Management of DLs involves not only the control of acute bleeding but also the prevention of recurrent bleeding. In this case, the patient was successfully treated with adrenaline injection and hemoclipping, but in some patients, other techniques are needed such as argon plasma coagulation, electrocoagulation, and embolization [7]. In severe cases, surgery may be necessary to control the bleeding and remove the affected tissue [7-9]. Endoscopic techniques have proven to be effective in treating GI DLs. In fact, there is a suggestion that these methods are more beneficial and successful than injection therapy when it comes to achieving long-lasting hemostasis for hemorrhaging DLs [10].

Overall, the outcome for patients with DLs is good if the condition is promptly diagnosed and treated. The prognosis for acute GI bleeding caused by DLs tends to be more favorable when contrasted with acute bleeding from gastric or duodenal ulcer [11].

## Conclusions

This case report emphasizes the importance of considering DLs as a potential cause of severe upper GI bleeding in pediatric patients. Early diagnosis through EGD and prompt intervention can effectively control bleeding and lead to favorable outcomes. It is important to emphasize the significance of early recognition and intervention in cases of DLs, especially in pediatric patients. DLs can cause life-threatening bleeding if left untreated. Additional investigation is required to enhance our comprehension of the occurrence, causes, and most effective approaches to managing DLs in the pediatric population.
